# Supplementary material for: Comparison of Fluid Replacement with Sterofundin ISO® vs. Deltajonin® in Infants Undergoing Craniofacial Surgery—A Retrospective Study
Source: J Clin Med. 2023 Oct 8;12(19):6404. doi: 10.3390/jcm12196404 (PMC10573171; doi:10.3390/jcm12196404)
Supplement: Supplementary file 1 [file jcm-12-06404-s001.zip › jcm-2604404-supplementary.pdf]

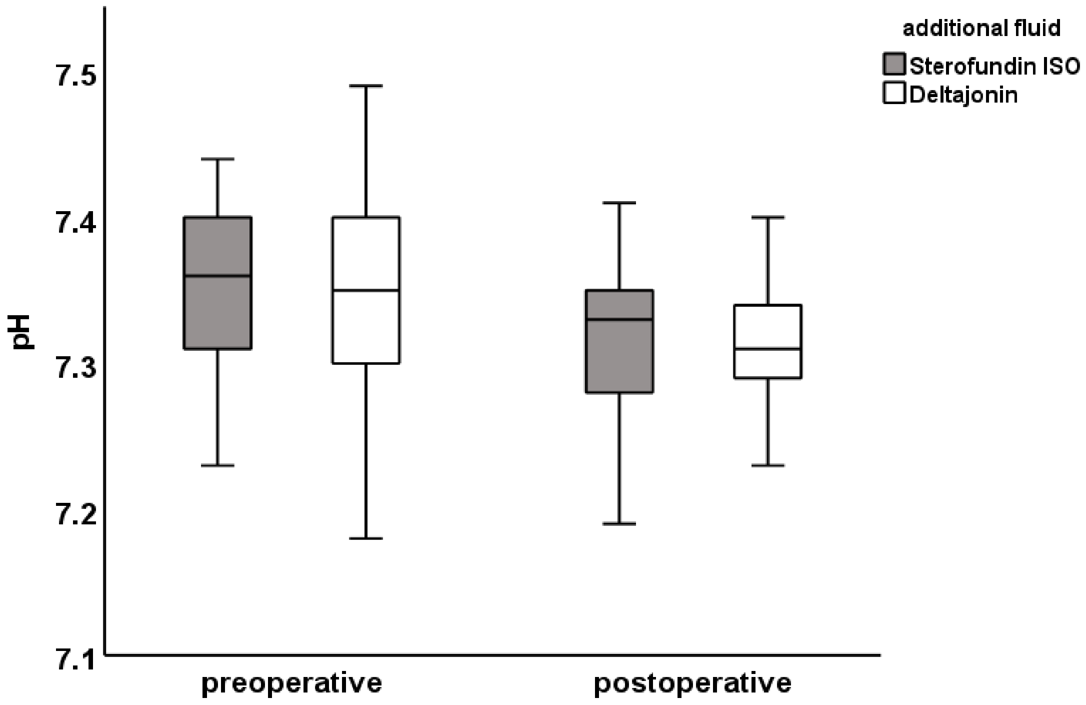

Figure S1. pH values pre- and postoperative. Blood samples were measured with an arterial blood gas analyzer right before and at the end of surgery. No Difference pre- or postoperative between Sterofundin ISO Group and Deltajonin Group; data are presented as mean ± SD.

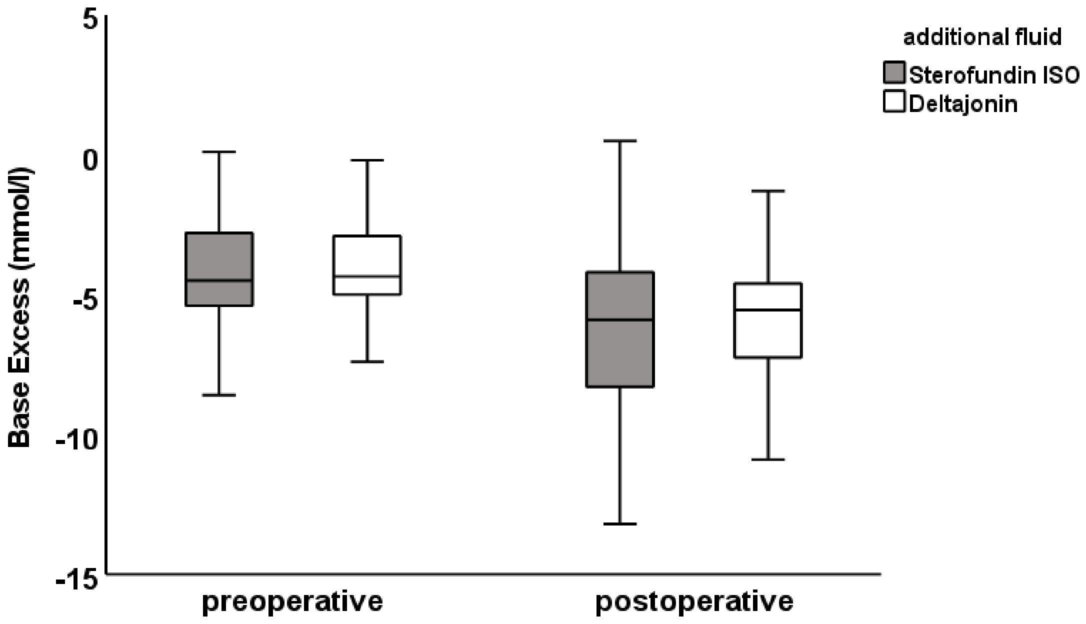

Figure S2. Base Excess values pre- and postoperative. Blood samples were measured with an arterial blood gas analyzer right before and at the end of surgery. No Difference pre- or postoperative between Sterofundin ISO Group and Deltajonin Group; data are presented as mean ± SD.

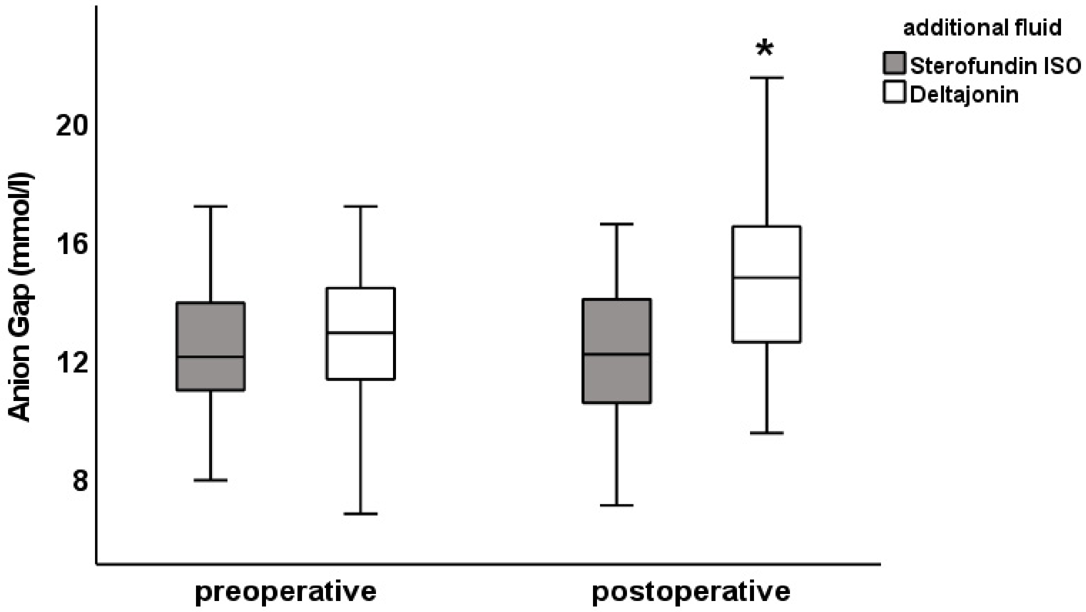

Figure S3. Anion Gap values pre- and postoperative. Anion Gap was calculated with [AG]= [Na+] + [K+] – [Cl-] – [Bic-]. Preoperative: Similar Anion Gap values. Postoperative: Difference between Sterofundin ISO Group and Deltajonin Group; data are presented as mean ± SD; * $p<0.05$ vs. Sterofundin ISO.

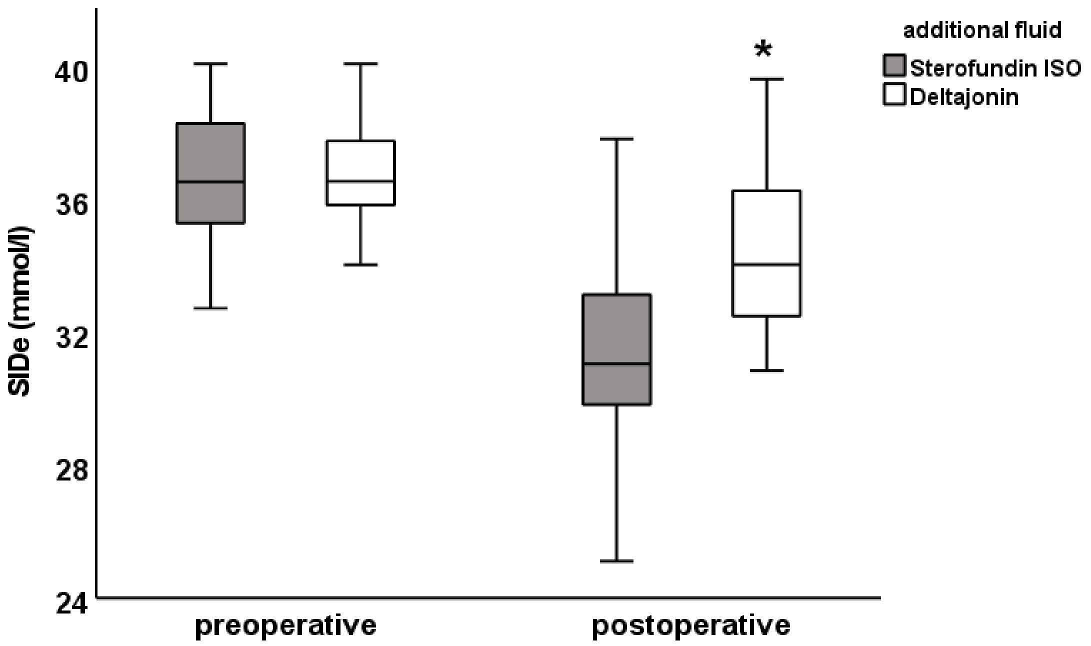

Figure S4. SIDe values pre- and postoperative. SIDe was calculated with [SIDe]= [A-] + [Bic]. Preoperative: Similar SIDe values. Postoperative: Difference between Sterofundin ISO Group and Deltajonin Group; data are presented as mean ± SD; * $p<0.05$ vs. Sterofundin ISO.

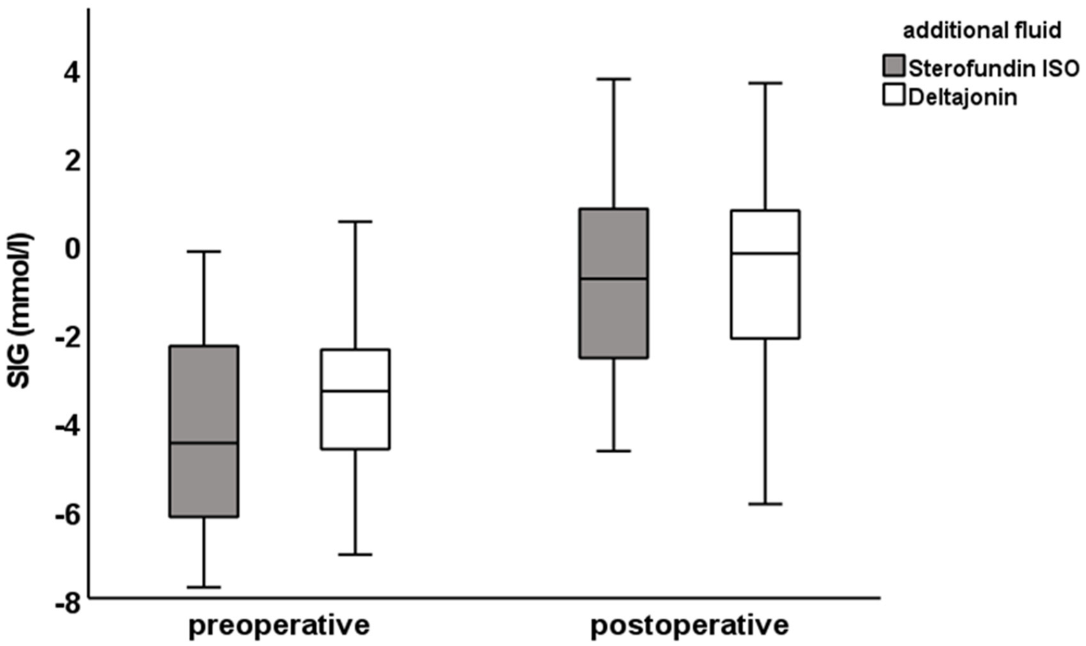

Figure S5. SIG values pre- and postoperative. SIG was calculated with [SIG]=[SIDa]-[SIDe]. No Difference pre- or postoperative between Sterofundin ISO Group and Deltajonin Group; data are presented as mean ± SD.

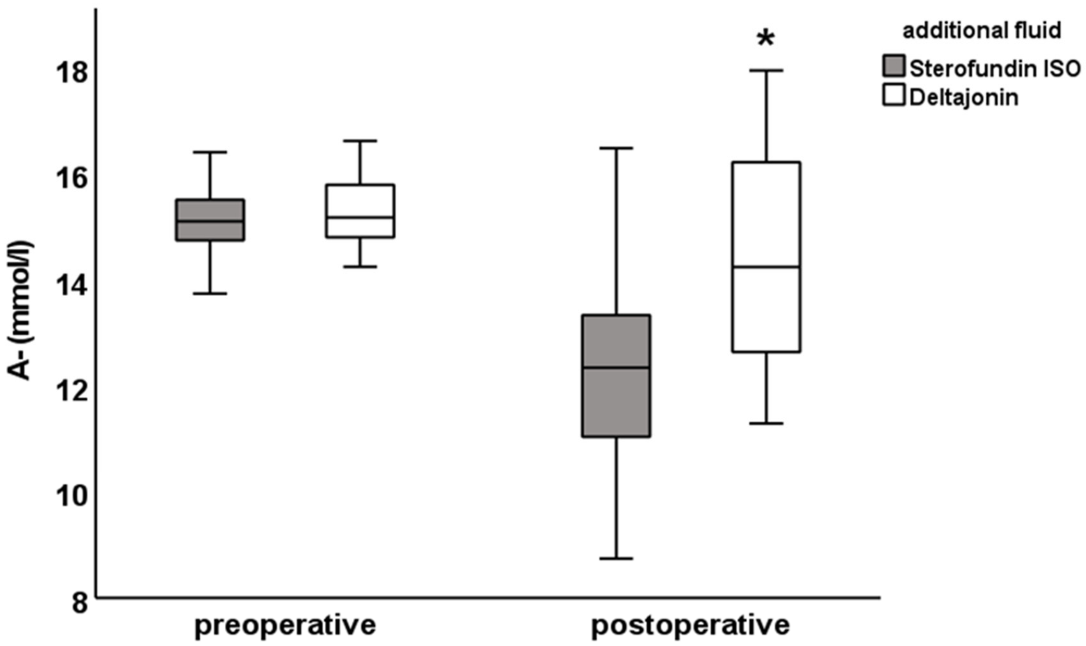

Figure S6. A- values pre- and postoperative. A- was calculated with [A-]= [albumin x (0.123 x pH – 0.631)] + [phosphate x (0.309 x pH – 0.469)]. Preoperative: Similar A- values. Postoperative: Difference between Sterofundin Iso Group and Deltajonin Group; data are presented as mean ± SD; * $p<0.05$ vs. Sterofundin Iso.

Table S1. Standard values.

Na^{+}-concentration	135-145 mmol/L
Cl^{-}-concentration	96-111 mmol/L
K^{+}-concentration	3.5-5.0 mmol/L
Mg^{++}	0.49-1.10 mmol/L
Ca^{++}	2.17-2.66 mmol/L
Lactate	5-20 mg/dL
Albumin (s)	37-51 g/L
Hemoglobin	11.1-14.3 g/dL
Phosphate	1.0-2.15 mmol/L
pH arteriell	7.37-7.45
pCO_2	35-45 mmHg
Bicarbonate	21-26 mmol/L
Base Excess	-2 – 3 mmol/L
Anionen Gap	12 mmol/L
SID_a	40 bzw. 47 mmol/L
SID_e	39 mmol/L
A^{-}	15 mmol/L

Table S2 Additional patient data.

ID	sex	age [months]	weight [kg]	duration of surgery [min]	ICU stay [days]	diagnosis	surgical technique	infusion
1	m	9	9	195	1	Nonsyndromic Sagittal Craniosynostisis (NSC)	Modified pi-procedure	Sterofundin ISO®
2	f	8	7	90	1	Nonsyndromic Sagittal Craniosynostisis (NSC)	Extended strip craniectomy with parietal osteotomies ("barrel staving")	Sterofundin ISO®
3	m	6	9	90	1	Nonsyndromic Sagittal Craniosynostisis (NSC)	Extended strip craniectomy with parietal osteotomies ("barrel staving")	Sterofundin ISO®
4	m	5	6,2	105	1	Nonsyndromic Sagittal Craniosynostisis (NSC)	Extended strip craniectomy with parietal osteotomies ("barrel staving")	Sterofundin ISO®
5	m	11	9,5	100	1	Nonsyndromic Sagittal Craniosynostisis (NSC)	Modified pi-procedure	Sterofundin ISO®
6	m	6	6,8	90	1	Nonsyndromic Sagittal Craniosynostisis (NSC)	Extended strip craniectomy with parietal osteotomies ("barrel staving")	Sterofundin ISO®
7	m	15	8,5	70	1	Nonsyndromic Sagittal Craniosynostisis (NSC)	Modified pi-procedure	Sterofundin ISO®
8	f	6	8	120	1	Nonsyndromic Sagittal Craniosynostisis (NSC)	Extended strip craniectomy with parietal osteotomies ("barrel staving")	Sterofundin ISO®
9	m	5	8	120	1	Nonsyndromic Sagittal Craniosynostisis (NSC)	Extended strip craniectomy with parietal osteotomies ("barrel staving")	Sterofundin ISO®
10	m	4	7,5	90	1	Nonsyndromic Sagittal Craniosynostisis (NSC)	Extended strip craniectomy with parietal osteotomies ("barrel staving")	Sterofundin ISO®
11	m	6	9	120	1	Nonsyndromic Sagittal Craniosynostisis (NSC)	Extended strip craniectomy with parietal osteotomies ("barrel staving")	Sterofundin ISO®
12	m	7	7	100	1	Nonsyndromic Sagittal Craniosynostisis (NSC)	Extended strip craniectomy with parietal osteotomies ("barrel staving")	Sterofundin ISO®
13	m	4	6,5	90	1	Nonsyndromic Sagittal Craniosynostisis (NSC)	Extended strip craniectomy with parietal osteotomies ("barrel staving")	Sterofundin ISO®
14	m	6	6,5	180	1	Nonsyndromic Sagittal Craniosynostisis (NSC)	Extended strip craniectomy with parietal osteotomies ("barrel staving")	Sterofundin ISO®
15	m	5	7	120	1	Nonsyndromic Sagittal Craniosynostisis (NSC)	Extended strip craniectomy with parietal osteotomies ("barrel staving")	Sterofundin ISO®
16	m	5	7,4	150	1	Nonsyndromic Sagittal Craniosynostisis (NSC)	Modified pi-procedure	Sterofundin ISO®
17	m	8	9,2	120	1	Nonsyndromic Sagittal Craniosynostisis (NSC)	Extended strip craniectomy with parietal osteotomies ("barrel staving")	Sterofundin ISO®
18	m	6	8	120	1	Nonsyndromic Sagittal Craniosynostisis (NSC)	Extended strip craniectomy with parietal osteotomies ("barrel staving")	Sterofundin ISO®
19	f	5	5,7	120	1	Nonsyndromic Sagittal Craniosynostisis (NSC)	Extended strip craniectomy with parietal osteotomies ("barrel staving")	Sterofundin ISO®
20	f	6	8,9	120	1	Nonsyndromic Sagittal Craniosynostisis (NSC)	Extended strip craniectomy with parietal osteotomies ("barrel staving")	Sterofundin ISO®
21	m	4	7,3	100	1	Nonsyndromic Sagittal Craniosynostisis (NSC)	Extended strip craniectomy with parietal osteotomies ("barrel staving")	Sterofundin ISO®
22	m	5	7,1	130	1	Nonsyndromic Sagittal Craniosynostisis (NSC)	Extended strip craniectomy with parietal osteotomies ("barrel staving")	Sterofundin ISO®
23	f	5	8,2	120	1	Nonsyndromic Sagittal Craniosynostisis (NSC)	Extended strip craniectomy with parietal osteotomies ("barrel staving")	Sterofundin ISO®
24	m	5	9	135	1	Nonsyndromic Sagittal Craniosynostisis (NSC)	Extended strip craniectomy with parietal osteotomies ("barrel staving")	Sterofundin ISO®
25	f	14	10	135	1	Nonsyndromic Sagittal Craniosynostisis (NSC)	Modified pi-procedure	Sterofundin ISO®
26	f	7	7,3	120	1	Nonsyndromic Sagittal Craniosynostisis (NSC)	Extended strip craniectomy with parietal osteotomies ("barrel staving")	Sterofundin ISO®
27	m	4,5	9	90	1	Nonsyndromic Sagittal Craniosynostisis (NSC)	Extended strip craniectomy with parietal osteotomies ("barrel staving")	Sterofundin ISO®
28	m	6	6,7	115	1	Nonsyndromic Sagittal Craniosynostisis (NSC)	Extended strip craniectomy with parietal osteotomies ("barrel staving")	Sterofundin ISO®
29	f	5	7,1	120	1	Nonsyndromic Sagittal Craniosynostisis (NSC)	Extended strip craniectomy with parietal osteotomies ("barrel staving")	Sterofundin ISO®
30	f	6	8	85	1	Nonsyndromic Sagittal Craniosynostisis (NSC)	Extended strip craniectomy with parietal osteotomies ("barrel staving")	Sterofundin ISO®
31	f	5	7	85	1	Nonsyndromic Sagittal Craniosynostisis (NSC)	Extended strip craniectomy with parietal osteotomies ("barrel staving")	Sterofundin ISO®
32	m	4,5	6,1	90	1	Nonsyndromic Sagittal Craniosynostisis (NSC)	Extended strip craniectomy with parietal osteotomies ("barrel staving")	Sterofundin ISO®
33	f	5	7	90	1	Nonsyndromic Sagittal Craniosynostisis (NSC)	Extended strip craniectomy with parietal osteotomies ("barrel staving")	Sterofundin ISO®
34	f	6	8	90	1	Nonsyndromic Sagittal Craniosynostisis (NSC)	Extended strip craniectomy with parietal osteotomies ("barrel staving")	Sterofundin ISO®
35	m	22	12,5	150	1	Nonsyndromic Sagittal Craniosynostisis (NSC)	Modified pi-procedure	Sterofundin ISO®
36	m	4,5	5,8	80	1	Nonsyndromic Sagittal Craniosynostisis (NSC)	Extended strip craniectomy with parietal osteotomies ("barrel staving")	Sterofundin ISO®
37	m	12	9	90	1	Nonsyndromic Sagittal Craniosynostisis (NSC)	Modified pi-procedure	Sterofundin ISO®
38	f	10	9	135	1	Nonsyndromic Sagittal Craniosynostisis (NSC)	Modified pi-procedure	Sterofundin ISO®
39	m	5	8,2	90	1	Nonsyndromic Sagittal Craniosynostisis (NSC)	Extended strip craniectomy with parietal osteotomies ("barrel staving")	Sterofundin ISO®
40	f	5	7,9	70	1	Nonsyndromic Sagittal Craniosynostisis (NSC)	Extended strip craniectomy with parietal osteotomies ("barrel staving")	Sterofundin ISO®
41	f	9	8	115	1	Nonsyndromic Sagittal Craniosynostisis (NSC)	Extended strip craniectomy with parietal osteotomies ("barrel staving")	Sterofundin ISO®
42	m	7	7,7	90	1	Nonsyndromic Sagittal Craniosynostisis (NSC)	Extended strip craniectomy with parietal osteotomies ("barrel staving")	Sterofundin ISO®
43	f	6	7	80	1	Nonsyndromic Sagittal Craniosynostisis (NSC)	Extended strip craniectomy with parietal osteotomies ("barrel staving")	Sterofundin ISO®
44	m	7	8	90	1	Nonsyndromic Sagittal Craniosynostisis (NSC)	Extended strip craniectomy with parietal osteotomies ("barrel staving")	Sterofundin ISO®
45	m	7	8,1	110	1	Nonsyndromic Sagittal Craniosynostisis (NSC)	Extended strip craniectomy with parietal osteotomies ("barrel staving")	Sterofundin ISO®
46	f	7	7	90	1	Nonsyndromic Sagittal Craniosynostisis (NSC)	Extended strip craniectomy with parietal osteotomies ("barrel staving")	Sterofundin ISO®
47	m	5	7,1	70	1	Nonsyndromic Sagittal Craniosynostisis (NSC)	Extended strip craniectomy with parietal osteotomies ("barrel staving")	Sterofundin ISO®
48	m	4	6,9	60	1	Nonsyndromic Sagittal Craniosynostisis (NSC)	Extended strip craniectomy with parietal osteotomies ("barrel staving")	Sterofundin ISO®
49	m	7	9	190	1	Nonsyndromic Sagittal Craniosynostisis (NSC)	Extended strip craniectomy with parietal osteotomies ("barrel staving")	Sterofundin ISO®
50	m	5	7,7	90	1	Nonsyndromic Sagittal Craniosynostisis (NSC)	Extended strip craniectomy with parietal osteotomies ("barrel staving")	Sterofundin ISO®
51	m	5	8,5	120	1	Nonsyndromic Sagittal Craniosynostisis (NSC)	Extended strip craniectomy with parietal osteotomies ("barrel staving")	Deltajonin®
52	f	9	7,5	90	1	Nonsyndromic Sagittal Craniosynostisis (NSC)	Modified pi-procedure	Deltajonin®
53	m	7	8,6	89	1	Nonsyndromic Sagittal Craniosynostisis (NSC)	Extended strip craniectomy with parietal osteotomies ("barrel staving")	Deltajonin®
54	m	14	10	180	1	Nonsyndromic Sagittal Craniosynostisis (NSC)	Modified pi-procedure	Deltajonin®
55	m	4	7,5	60	1	Nonsyndromic Sagittal Craniosynostisis (NSC)	Extended strip craniectomy with parietal osteotomies ("barrel staving")	Deltajonin®

56	f	12	11	87	1	Nonsyndromic Sagittal Craniosynostisis (NSC)	Modified pi-procedure	Deltajonin®
57	f	4,5	6,5	90	1	Nonsyndromic Sagittal Craniosynostisis (NSC)	Extended strip craniectomy with parietal osteotomies ("barrel staving")	Deltajonin®
58	m	7	7,5	60	1	Nonsyndromic Sagittal Craniosynostisis (NSC)	Extended strip craniectomy with parietal osteotomies ("barrel staving")	Deltajonin®
59	m	6	8,8	90	1	Nonsyndromic Sagittal Craniosynostisis (NSC)	Extended strip craniectomy with parietal osteotomies ("barrel staving")	Deltajonin®
60	m	9	10,8	80	1	Nonsyndromic Sagittal Craniosynostisis (NSC)	Modified pi-procedure	Deltajonin®
61	m	5	7	90	1	Nonsyndromic Sagittal Craniosynostisis (NSC)	Extended strip craniectomy with parietal osteotomies ("barrel staving")	Deltajonin®
62	m	8	9	105	1	Nonsyndromic Sagittal Craniosynostisis (NSC)	Extended strip craniectomy with parietal osteotomies ("barrel staving")	Deltajonin®
63	m	4,5	8	90	1	Nonsyndromic Sagittal Craniosynostisis (NSC)	Extended strip craniectomy with parietal osteotomies ("barrel staving")	Deltajonin®
64	m	8	7,8	60	1	Nonsyndromic Sagittal Craniosynostisis (NSC)	Extended strip craniectomy with parietal osteotomies ("barrel staving")	Deltajonin®
65	f	8	8,5	120	1	Nonsyndromic Sagittal Craniosynostisis (NSC)	Extended strip craniectomy with parietal osteotomies ("barrel staving")	Deltajonin®
66	m	5	6,5	90	1	Nonsyndromic Sagittal Craniosynostisis (NSC)	Extended strip craniectomy with parietal osteotomies ("barrel staving")	Deltajonin®
67	m	5	6,1	75	1	Nonsyndromic Sagittal Craniosynostisis (NSC)	Extended strip craniectomy with parietal osteotomies ("barrel staving")	Deltajonin®
68	f	11	8,5	225	1	Nonsyndromic Sagittal Craniosynostisis (NSC)	Modified pi-procedure	Deltajonin®
69	m	7	9,5	88	1	Nonsyndromic Sagittal Craniosynostisis (NSC)	Extended strip craniectomy with parietal osteotomies ("barrel staving")	Deltajonin®
70	m	6	7,5	90	1	Nonsyndromic Sagittal Craniosynostisis (NSC)	Extended strip craniectomy with parietal osteotomies ("barrel staving")	Deltajonin®
71	f	9	8,1	70	1	Nonsyndromic Sagittal Craniosynostisis (NSC)	Extended strip craniectomy with parietal osteotomies ("barrel staving")	Deltajonin®
72	m	9	8	90	1	Nonsyndromic Sagittal Craniosynostisis (NSC)	Extended strip craniectomy with parietal osteotomies ("barrel staving")	Deltajonin®
73	f	11	9,2	70	1	Nonsyndromic Sagittal Craniosynostisis (NSC)	Modified pi-procedure	Deltajonin®
74	f	8	9,7	60	1	Nonsyndromic Sagittal Craniosynostisis (NSC)	Extended strip craniectomy with parietal osteotomies ("barrel staving")	Deltajonin®
75	f	9	12	60	1	Nonsyndromic Sagittal Craniosynostisis (NSC)	Extended strip craniectomy with parietal osteotomies ("barrel staving")	Deltajonin®
76	m	7	9,2	70	1	Nonsyndromic Sagittal Craniosynostisis (NSC)	Extended strip craniectomy with parietal osteotomies ("barrel staving")	Deltajonin®
77	f	6	7	90	1	Nonsyndromic Sagittal Craniosynostisis (NSC)	Extended strip craniectomy with parietal osteotomies ("barrel staving")	Deltajonin®
78	m	6	7	120	1	Nonsyndromic Sagittal Craniosynostisis (NSC)	Extended strip craniectomy with parietal osteotomies ("barrel staving")	Deltajonin®
79	m	5	7	40	1	Nonsyndromic Sagittal Craniosynostisis (NSC)	Extended strip craniectomy with parietal osteotomies ("barrel staving")	Deltajonin®
80	m	5	8,8	70	1	Nonsyndromic Sagittal Craniosynostisis (NSC)	Extended strip craniectomy with parietal osteotomies ("barrel staving")	Deltajonin®
81	m	5,5	6,3	75	1	Nonsyndromic Sagittal Craniosynostisis (NSC)	Extended strip craniectomy with parietal osteotomies ("barrel staving")	Deltajonin®
82	m	11	10	70	1	Nonsyndromic Sagittal Craniosynostisis (NSC)	Modified pi-procedure	Deltajonin®
83	f	9	7,8	105	1	Nonsyndromic Sagittal Craniosynostisis (NSC)	Extended strip craniectomy with parietal osteotomies ("barrel staving")	Deltajonin®
84	f	13	11	60	1	Nonsyndromic Sagittal Craniosynostisis (NSC)	Extended strip craniectomy with parietal osteotomies ("barrel staving")	Deltajonin®
85	f	7,1	6	105	1	Nonsyndromic Sagittal Craniosynostisis (NSC)	Extended strip craniectomy with parietal osteotomies ("barrel staving")	Deltajonin®
86	f	7	8,1	90	1	Nonsyndromic Sagittal Craniosynostisis (NSC)	Extended strip craniectomy with parietal osteotomies ("barrel staving")	Deltajonin®
87	m	6	10,73	90	1	Nonsyndromic Sagittal Craniosynostisis (NSC)	Extended strip craniectomy with parietal osteotomies ("barrel staving")	Deltajonin®
88	m	7	9,5	60	1	Nonsyndromic Sagittal Craniosynostisis (NSC)	Extended strip craniectomy with parietal osteotomies ("barrel staving")	Deltajonin®
89	m	5	7,5	70	1	Nonsyndromic Sagittal Craniosynostisis (NSC)	Extended strip craniectomy with parietal osteotomies ("barrel staving")	Deltajonin®
90	m	6	10,7	70	1	Nonsyndromic Sagittal Craniosynostisis (NSC)	Extended strip craniectomy with parietal osteotomies ("barrel staving")	Deltajonin®
91	m	8	9,5	120	1	Nonsyndromic Sagittal Craniosynostisis (NSC)	Extended strip craniectomy with parietal osteotomies ("barrel staving")	Deltajonin®
92	f	3,5	5,5	90	1	Nonsyndromic Sagittal Craniosynostisis (NSC)	Extended strip craniectomy with parietal osteotomies ("barrel staving")	Deltajonin®
93	m	10	10,5	120	1	Nonsyndromic Sagittal Craniosynostisis (NSC)	Extended strip craniectomy with parietal osteotomies ("barrel staving")	Deltajonin®
95	f	5	5,5	120	1	Nonsyndromic Sagittal Craniosynostisis (NSC)	Extended strip craniectomy with parietal osteotomies ("barrel staving")	Deltajonin®
96	m	7	7,5	60	1	Nonsyndromic Sagittal Craniosynostisis (NSC)	Extended strip craniectomy with parietal osteotomies ("barrel staving")	Deltajonin®
97	m	5	8	60	1	Nonsyndromic Sagittal Craniosynostisis (NSC)	Extended strip craniectomy with parietal osteotomies ("barrel staving")	Deltajonin®
98	m	12	10,2	90	1	Nonsyndromic Sagittal Craniosynostisis (NSC)	Modified pi-procedure	Deltajonin®
99	m	7	7,5	90	1	Nonsyndromic Sagittal Craniosynostisis (NSC)	Extended strip craniectomy with parietal osteotomies ("barrel staving")	Deltajonin®
100	m	8	10	60	1	Nonsyndromic Sagittal Craniosynostisis (NSC)	Extended strip craniectomy with parietal osteotomies ("barrel staving")	Deltajonin®
